# Reply to Haq et al. Comment on “Leivaditis et al. Artificial Intelligence in Cardiac Surgery: Transforming Outcomes and Shaping the Future. *Clin. Pract.* 2025, *15*, 17”

**DOI:** 10.3390/clinpract15110196

**Published:** 2025-10-27

**Authors:** Vasileios Leivaditis, Eleftherios Beltsios, Athanasios Papatriantafyllou, Konstantinos Grapatsas, Francesk Mulita, Nikolaos Kontodimopoulos, Nikolaos G. Baikoussis, Levan Tchabashvili, Konstantinos Tasios, Ioannis Maroulis, Manfred Dahm, Efstratios Koletsis

**Affiliations:** 1 Department of Cardiothoracic and Vascular Surgery, WestpfalzKlinikum, 67655 Kaiserslautern, Germany; vleivaditis@westpfalz-klinikum.de (V.L.); apapatriantafy@westpfalz-klinikum.de (A.P.); mdahm@westpfalz-klinikum.de (M.D.); 2 Department of Anesthesiology and Intensive Care, Hannover Medical School, 30625 Hannover, Germany; beltsios.eleftherios@mh-hannover.de; 3 Department of Thoracic Surgery and Thoracic Endoscopy, Ruhrlandklinik, West German Lung Center, University Hospital Essen, University Duisburg-Essen, 45141 Essen, Germany; grapatsaskostas@gmail.com; 4 Department of General Surgery, General University Hospital of Patras, 26504 Patras, Greece; levatcha@pgnp.gr (L.T.); ceid4551@ac.upatras.gr (K.T.); 5 Department of Economics and Sustainable Development, Harokopio University, 17778 Athens, Greece; nkontodi@otenet.gr; 6 Department of Cardiac Surgery, Ippokrateio General Hospital of Athens, 11527 Athens, Greece; nikolaos.baikoussis@gmail.com; 7 Department of Cardiothoracic Surgery, General University Hospital of Patras, 26504 Patras, Greece; ekoletsis@med.upatras.gr

We thank Haq and Khan for their thoughtful commentary [1] on our review of artificial intelligence (AI) in cardiac surgery [2]. We fully agree that advancing AI integration in this field requires not only technological innovation but also robust strategies for data quality, workflow integration, ethical governance, and interdisciplinary collaboration.

## 1. Data Quality and Generalizability

As emphasized, the effectiveness of AI models is contingent on the diversity and representativeness of their training datasets. Our review highlighted similar concerns and supports the authors’ call for prospective, multicenter validation efforts. Frameworks such as DECIDE-AI provide timely guidance for ensuring that models meet standards of transparency and generalizability [3].

## 2. Clinical Integration and Workflow Challenges

Resistance to adopting AI often stems from usability and interoperability issues within existing clinical workflows. We concur that early involvement of clinicians, user-centered design, and iterative feedback loops are pivotal to achieving meaningful and sustainable implementation of AI-driven decision-support systems [4].

## 3. Ethical Accountability and Algorithmic Bias

The concerns raised about accountability and algorithmic bias align with our conclusions that AI must complement—not replace—human judgment. Future research should prioritize explainable AI, equitable data representation, and strong medico-legal frameworks to build trust in high-stakes environments such as cardiac surgery [5].

## 4. Interdisciplinary Collaboration

We echo the recommendation to strengthen collaboration among engineers, clinicians, ethicists, regulators, and patient advocates. Innovative tools, including virtual and augmented reality, exemplify the potential of interdisciplinary approaches to enhance preoperative planning and intraoperative guidance [6].

In summary, the dialog initiated by Haq and Khan underscores the importance of continuous discourse on equitable, explainable, and patient-centered AI. Progress in these domains will be key to realizing AI’s full promise in transforming cardiac surgical care.
